# Whole Number, Distribution and Co-Expression of Brn3 Transcription Factors in Retinal Ganglion Cells of Adult Albino and Pigmented Rats

**DOI:** 10.1371/journal.pone.0049830

**Published:** 2012-11-16

**Authors:** Francisco M. Nadal-Nicolás, Manuel Jiménez-López, Manuel Salinas-Navarro, Paloma Sobrado-Calvo, Juan J. Alburquerque-Béjar, Manuel Vidal-Sanz, Marta Agudo-Barriuso

**Affiliations:** 1 Unidad de Investigación. Hospital Universitario Virgen de la Arrixaca. Fundación para la Formación e Investigación Sanitarias de la Región de Murcia. IMIB, El Palmar, Murcia, Spain; 2 Departamento de Oftalmología, Facultad de Medicina, Universidad de Murcia, IMIB, Espinardo, Murcia, Spain; The University of Western Australia, Australia

## Abstract

The three members of the *Pou4f* family of transcription factors: *Pou4f1, Pou4f2, Pou4f3* (Brn3a, Brn3b and Brn3c, respectively) play, during development, essential roles in the differentiation and survival of sensory neurons. The purpose of this work is to study the expression of the three Brn3 factors in the albino and pigmented adult rat. Animals were divided into these groups: i) untouched; ii) fluorogold (FG) tracing from both superior colliculli; iii) FG-tracing from one superior colliculus; iv) intraorbital optic nerve transection or crush. All retinas were dissected as flat-mounts and subjected to single, double or triple immunohistofluorescence The total number of FG-traced, Brn3a, Brn3b, Brn3c or Brn3 expressing RGCs was automatically quantified and their spatial distribution assessed using specific routines. Brn3 factors were studied in the general RGC population, and in the intrinsically photosensitive (ip-RGCs) and ipsilateral RGC sub-populations. Our results show that: i) 70% of RGCs co- express two or three Brn3s and the remaining 30% express only Brn3a (26%) or Brn3b; ii) the most abundant Brn3 member is Brn3a followed by Brn3b and finally Brn3c; iii) Brn3 a-, b- or c- expressing RGCs are similarly distributed in the retina; iv) The vast majority of ip-RGCs do not express Brn3; v) The main difference between both rat strains was found in the population of ipsilateral-RGCs, which accounts for 4.2% and 2.5% of the total RGC population in the pigmented and albino strain, respectively. However, more ipsilateral-RGCs express Brn3 factors in the albino than in the pigmented rat; vi) RGCs that express only Brn3b and RGCs that co-express the three Brn3 members have the biggest nuclei; vii) After axonal injury the level of Brn3a expression in the surviving RGCs decreases compared to control retinas. Finally, this work strengthens the validity of Brn3a as a marker to identify and quantify rat RGCs.

## Introduction

The Brn3 family of transcription factors (Brn3a/*Pou4f1*, Brn3b/*Pou4f2*, Brn3c/*Pou4f3*) [1]–[3] are expressed by different sets of neurons of the trigeminal nuclei, dorsal root ganglia, inner ear and retina [4]. They are not needed for neuronal commitment but are crucial for neuronal differentiation and survival [5]–[10]. Each Brn3 is expressed in a characteristic spatiotemporal pattern that differs among each sensory organ [4], [5], [7], [9], [11]–[14]. In fact, in each of these organs a different Brn3 gene is the first to be expressed. This initial expression is followed by an orchestrated combinatorial expression that controls the neuronal differentiation and diversity of each sensory organ. In addition, it has been shown that Brn3a and Brn3b are functionally equivalent, thus during development Brn3a can replace Brn3b function if it is expressed during the Brn3b spatiotemporal window [15], [16].

In the retina, Brn3 transcription factors are exclusively expressed in retinal ganglion cells (RGCs). RGCs are the only retinal neurons that send their axons (forming the optic nerve) outside the eye to synapse with central targets: superior colliculi; lateral geniculate nucleus; intergeniculate nucleus; the dorsal, lateral and medial terminal nuclei, the olivary pretectal nuclei and the supraquiasmatic nuclei (reviewed in [17]). In rodents, mice and rats, the vast majority of RGCs project contralaterally to the superior colliculi [18]–[23]. RGCs convey information that is relevant to image-forming and non-image forming vision such as eye movement control, pupilary light reflex and circadian photo-entrainment. There are more than twenty types of RGCs which are not easily distinguished since up to date there are no subtype-specific molecular markers except for two of these subtypes, the intrinsically photosensitive RGCs (ip-RGCs) and the RGCs that selectively respond to upward motion [24]. ip-RGCs express a specific photopigment, melanopsin [25], [26], and are responsible of non-image forming visual functions, although recently it has been shown that they are implied as well in image forming vision [27], [28].

The role of Brn3 factors in the development, differentiation, morphology and function of RGCs has been thoroughly studied in mice [6], [7], [12], [29]–[37]. During retinal development, the first Brn3 member to be expressed in RGCs is Brn3b, followed one day later by Brn3a and finally by Brn3c [34]. Thus, 80% of the developing RGCs express Brn3b in combination or not with Brn3a and Brn3c [5], [35]. Brn3b is essential for RGC differentiation, survival and axon pathfinding [33], Brn3c seems to be required for ipsilateral projections [6] and Brn3a is part of a regulatory network that controls RGC dendritic stratification [36].

In rat, all the genetic analyses carried out in mouse are not feasible and thus the role of Brn3 factors during rat development remains to be addressed.

As in mouse, [38] adult rat RGCs express Brn3a, though in rat this population is higher than in mouse [39]. Furthermore, in rat, Brn3a expression is maintained in those RGCs that although being injured, are still alive [40]. Both features make Brn3a immunodetection an excellent tool to detect and quantify RGCs in this species.

Albinism i.e. the lack of melanin causes a long list of abnormalities, which in the visual system are: an impaired visual acuity and defects in the crossing of the retinofugal projections and optokinetic nystagmus [41]–[43], reviewed in [44].

Little is known about Brn3b [45] and Brn3c expression in the adult rat retina or about their response to axonal injury. Because of this and because, to date, there is not a comprehensive study of the expression relationship among the three Brn3 members in this species, the general goal of this work is to characterize the population of Brn3 a-, b- and c- expressing RGCs in two rat strains, one albino and one pigmented. Both strains were compared to elucidate whether albinism has, as well, an effect on the number and distribution of Brn3^+^RGCs. Finally, the expression of the three Brn3 factors was studied in axotomized retinas.

## Results

### Brn3 Co-expression

**Figure 1:**
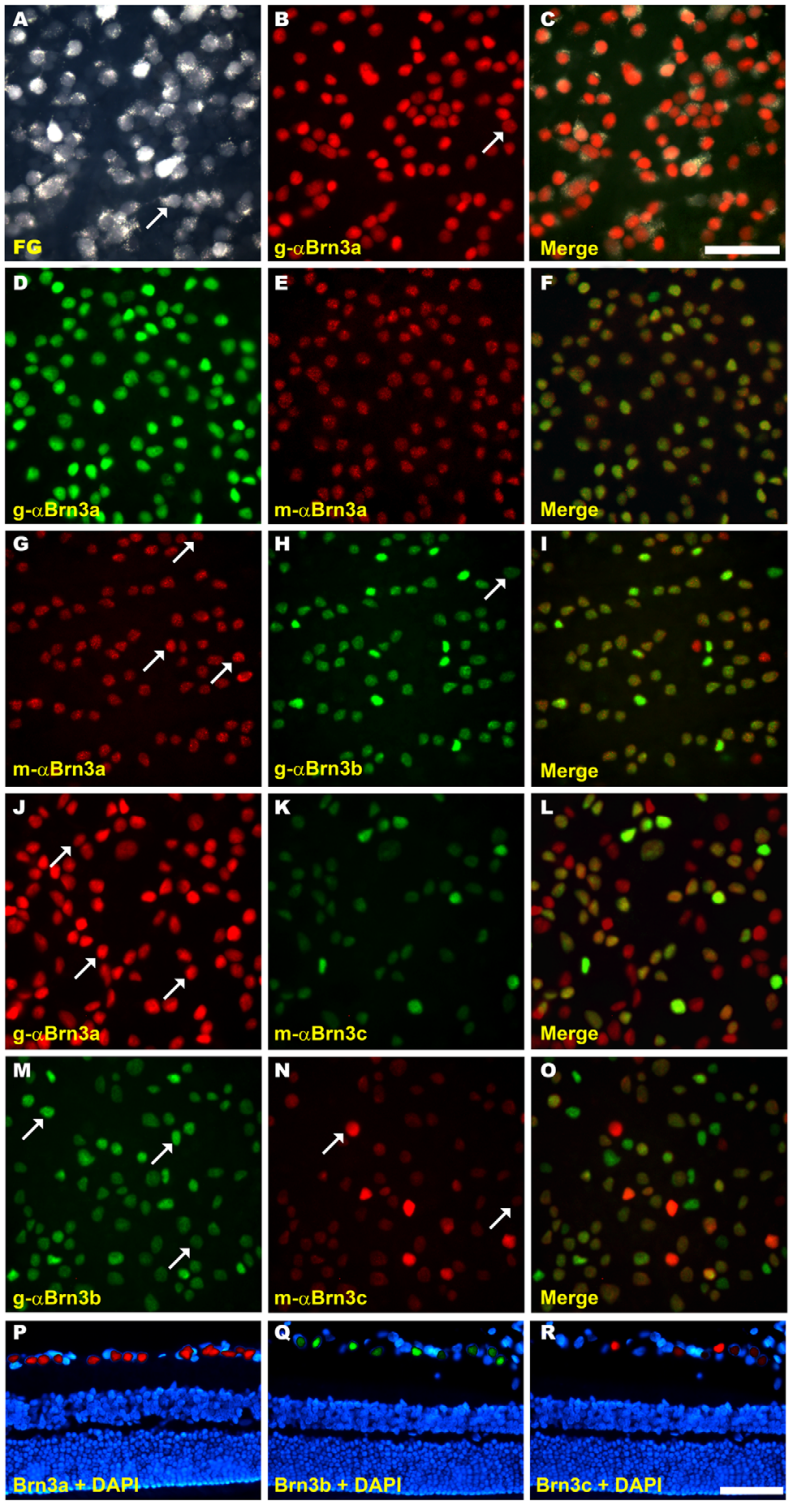
Co-expression of Brn3 transcription factors in albino and pigmented rats. A–O: Magnifications from SD flat mounted retinas in which FG and Brn3a (**A–C**); Brn3a detected with two different antibodies -goat and mouse anti-Brn3a-(**D–F**); Brn3a and b (**G–H**), Brn3a and c (**J–L**) and Brn3b and c (**M–O**) have been double detected. In images like these taken from SD (albino) and PVG (pigmented) rat retinas the percentage of co-localization of each marker (see figure 2) was calculated. **P–R:** cross-sections from SD rat retinas, in which Brn3a (**P**), Brn3b (**Q**) and Brn3c (**R**) have been detected to show that these proteins are only expressed in the ganglion cell layer. In these images, all nuclei have been counterstained with DAPI. Arrows point to those RGCs that only express one of the Brn3s. *Bar*: 50 µm (C,R).

In albino rats the percentage of Brn3a^+^RGCs that are FG-traced coincides with previous reports (95.4%: 1,995 RGCs Brn3a and FG^+^out of 2,088 counted Brn3a^+^RGCs [39]). In pigmented rats this percentage is slightly higher (96.3%: 4,209 RGCs Brn3a and FG^+^out of 4,367 counted Brn3a^+^RGCs; **figure 1** **A–C**).

In the albino strain the percentage of FG^+^RGCs that express Brn3a is 92.2% [39], while in the pigmented one this percentage goes up to 96.4% (out of 4,365 FG^+^RGCs, 4,209 were Brn3a^+^as well).

To detect Brn3a we use a goat α-Brn3a antibody [39]. But because to assess Brn3a and Brn3b co-expression it was necessary to use a mouse α-Brn3a antibody which had not yet been tested, both α-Brn3a antibodies (goat and mouse) were double immunodetected to determine their level of co-detection (**Figure 1** **D–F**). Out of 2,644 (SD) and 2,681 (PVG) counted mαBrn3a^+^RGCs all were gαBrn3a^+^but no vice versa as 113 (SD) and 64 (PVG) RGCs were only gαBrn3a^+^. Thus, the gαBrn3a antibody detects a 4.1% and 2.3% more RGCs in SD and PVG retinas, respectively, than the mouse one. These differences were not significant (Mann-Whitney test, p = 0.623 in albino rats, p = 0.709 in pigmented ones).

**Figure 2:**
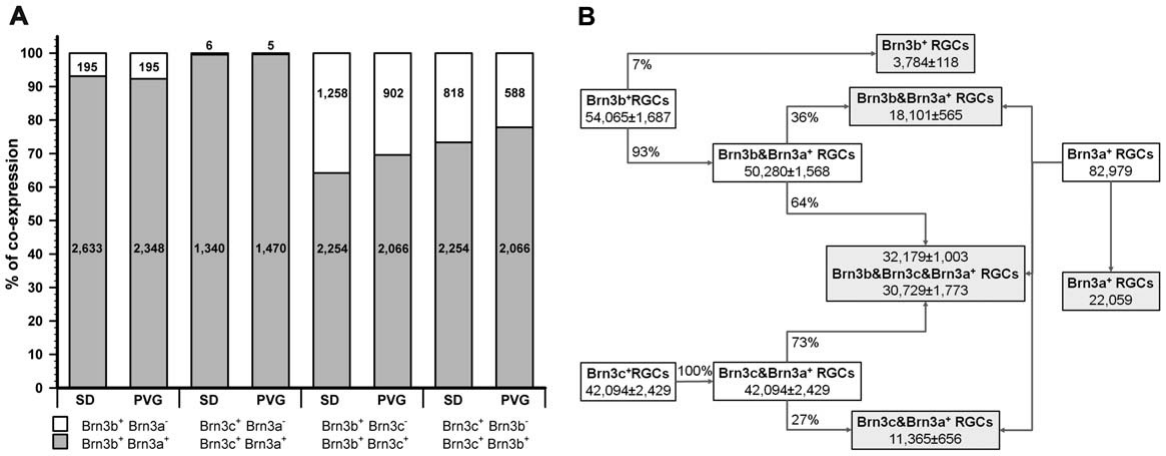
Brn3 co-expression in albino and pigmented rat retinas. **A:** Stacked-bar graph showing the percentage of Brn3 co-expression in both rat strains. In each bar is shown the number of cells counted. First and second bars: percentage of Brn3b^+^RGCs that express or not Brn3a. Third and fourth bars: percentage of Brn3c^+^RGCs that express or not Brn3a. Fifth and sixth bars: percentage of Brn3b^+^RGCs that express or not Brn3c. Seventh and eighth bars: percentage of Brn3c^+^RGCs that express or not Brn3b. Percentage was calculated considering 100% the total number of cells per marker (see results for further explanation). **B**: Inference of the total number of RGCs that express one, two or the three Brn3 members in the albino strain. These numbers were calculated based on the co-expression percentages and on the total number of RGCs (see table 1 and results). The final population for each marker combination is shown in the grey squares.

Images illustrating the double detection of Brn3a and b, Brn3a and c, and Brn3b and c are shown in **figure 1** **G–O** and the quantitative data in **figure 2A** **.** Co-expression percentages were calculated considering 100% the total number of RGCs expressing Brn3b or Brn3c. For example, co-expression of Brn3b and Brn3c in albino rats (fifth bar **figure 2A**): out of 3,512 counted Brn3b^+^RGCs, 2,254 were Brn3c positive and 1,258 were Brn3c negative. Thus 64% of Brn3b^+^RGCs also express Brn3c while 36% do not. This was done for all combinations, and the data show that: i) co-expression levels are very similar between both rat strains; ii) a higher percentage of Brn3c^+^RGCs express Brn3b (73.4% albino, 77.8% pigmented) than Brn3b^+^RGCs express Brn3c (64.2% albino, 69.6% pigmented); iii) practically all (99.6% albino, 99.7% pigmented) Brn3c^+^RGCs express Brn3a; iv) a small percentage of Brn3b^+^RGCs do not express Brn3a (6.9% albino, 7.7% pigmented), although these percentages might be overvalued because the mouse α-Brn3a detects less RGCs that the goat one.

**Table 1:** Total numbers and densities of Brn3 positive RGCs in albino and pigmented rats.

SD	Fluorogold	Brn3a	Brn3b	Brn3c	Brn3
RGC number Mean±SD	82,595±1,568	82,979±1,787	54,065±1,687	42,094±2,429	84,700±2,148
Area (mm^2^) Mean±SD	54.6±1.9		54.7±0.7		54.25±2.6
Density (RGCs/mm^2^) Mean±SD	1,512±63	1,519±59	987±38	769±53	1,565±106
n	6		9		8
PVG	Fluorogold	Brn3a	Brn3b	Brn3c	Brn3
RGC number Mean±SD	84,112±5,531	84,814±4,119	54,921±3,723	41,183±2,071	85,778±1,820
Area (mm^2^) Mean±SD	63.16±5.2		66.5±1.5		66.25±1.16
Density (RGCs/mm^2^) Mean±SD	1,316±57	1,331±129	826±156	619±31	1,291±46
n	12		8		8

In this table are shown the total numbers of the different RGC populations (FG-traced, Brn3a, Brn3b or Brn3c positive RGCs) in albino (SD) and pigmented (PVG) rats. In the right-most column is shown the total number of Brn3^+^RGCs counted when Brn3a, Brn3b and Brn3c were detected with the same fluorophore. As explained in methods, FG and Brn3a and Brn3b and Brn3c were detected in the same retinas. The area of each retina was measured allowing the calculation of the mean retinal density of each population. Data are shown as the mean ± standard deviation (SD). n = number of analyzed retinas.

In **figure 1** **P–R** is shown that the three Brn3 members are only expressed in the ganglion cell layer.

### Brn3 Expression by Intrinsically Photosensitive RGCs (ip-RGCs)

**Figure 3:**
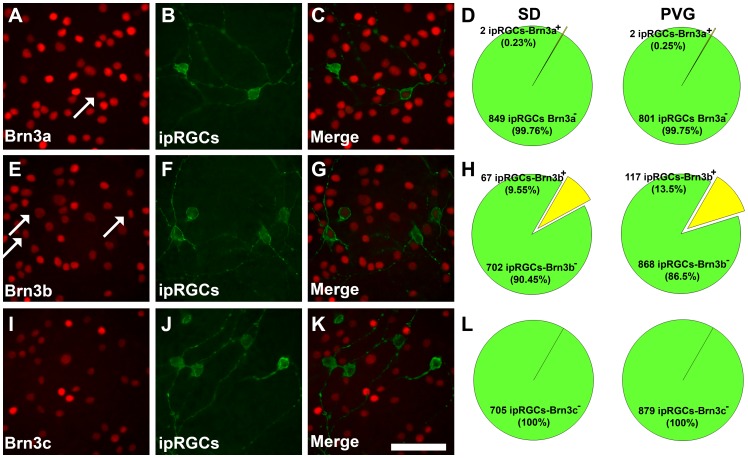
Expression of Brn3 transcription factors by intrinsically photosensitive RGCs (ip-RGCs). Magnifications from SD flat mounted retinas in which Brn3a and melanopsin (**A**–**C**), Brn3b and melanopsin (**E–G**) and Brn3c and melanopsin (**I–J**) have been double immunodetected. Arrows point to doubly labelled ip-RGCs. **D**, **H**, and **L:** Quantification of the number of ip-RGCs that express Brn3a, b or c in albino (left) and pigmented (right) rats. In these pies is shown the net number of counted ip-RGCs and the percentage of them that express a given Brn3 member. *Bars*: 50 µm (**K, R**). n = 4 retinas per marker and strain.

ip-RGCs are distinguished from the rest of the RGC population because they express melanopsin [25]. Thus, melanopsin and each of the Brn3 members were immunodetected in retinas from albino and pigmented animals to assess their level of co-expression (**Figure 3**). These results show that the vast majority of ip-RGCs do not express these transcription factors. In fact, in both strains none of them express Brn3c; Brn3a is expressed by a 0.23% (SD) and 0.25% (PVG) of them, and 9.55% in the albino and 13.6% in the pigmented strain express Brn3b.

### Total Number of RGCs in Albino and Pigmented Rats

Quantitative data are shown in **table 1**. In the pigmented strain, the whole number of Brn3^+^, Brn3a^+^and FG-traced RGCs is higher than in the albino strain, but this difference is not statistically significant (Mann Whitney test p = 0.673 for FG, p = 0.325 for Brn3a and p = 0.297 for Brn3). However, the total number of Brn3b and Brn3c positive RGCs is similar in both strains.

The total population of Brn3^+^RGCs (Brn3a+Brn3b+Brn3c triple immunodetection using the same fluorophore) is not the sum of the three Brn3s populations. In fact, is higher than the population of Brn3a^+^RGCs only by 1,721 (SD) and 924 (PVG) RGCs. This indicates, in agreement with the previous data, a high level of Brn3 co-expression.

Using these data (**table 1**) and the level of Brn3 co-expression (**Figure 2A**), the population of RGCs that express three, two or one Brn3 was inferred for the albino strain (**Figure 2B** **)** as follows: Out of the 54,065 Brn3b^+^RGCs, 93% express also Brn3a^+^(50,280); of these 64% express Brn3c and 36% do not. Thus 18,101 RGCs express Brn3b and Brn3a but do not express Brn3c and 32,179 express the three Brn3 members. Notice that the number of RGCs that express the three members varies slightly if the inference is done from the Brn3b or from the Brn3c population. Out of the total of Brn3b population 7% do not express Brn3a (3,784); out of these 64% would also express Brn3c but because all Brn3c^+^RGCs express Brn3a, these are probably RGCs that only express Brn3b. The same procedure was applied starting from the Brn3c population.

The number of RGCs expressing only Brn3a was calculated by subtracting from their total population (82,979) the RGCs-Brn3a and Brn3b and/or Brn3c positive. These calculations disclose that over 22,000 RGCs express only Brn3a (26.6% of the Brn3a population, 25.9% of the Brn3 population) and that around 31,500 express the three Brn3 members (37% of the Brn3 population). In addition 18,101 express Brn3a and Brn3b but no Brn3c (21.3%) and 11,365 express Brn3c and Brn3a but no Brn3b (13.4%). A small population of RGCs express only Brn3b (3,784, 4.5%), but as abovementioned part of these RGCs might be Brn3a positive and thus this percentage might be lower.

In conclusion, approximately a 35% of the RGCs express the three Brn3s, 35% two of them (a+b or a+c) and the remaining 30% one of them (a or b).

### RGC Spatial Distribution

**Figure 4:**
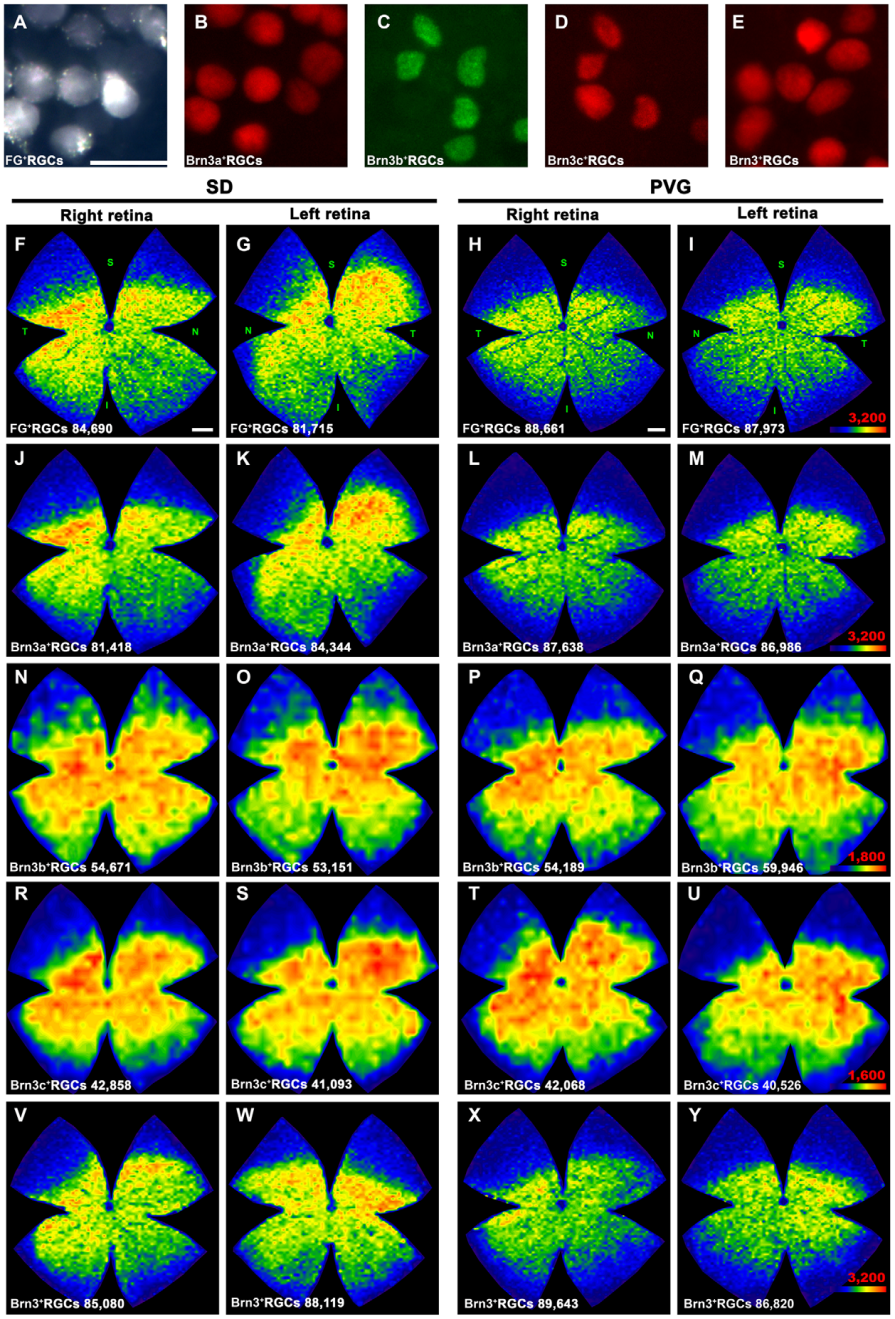
Spatial distribution of RGCs in albino and pigmented rats. **A–E**: magnifications from flat mounted retinas showing RGCs detected by FG tracing (**A**), Brn3a (**B**), Brn3b (**C**), Brn3c (**D**) and Brn3a+b+c immunodetection. **A, B** and **C, D** are images taken from the same retinal frame. In **E** the three Brn3 members were detected using the same fluorophore (Brn3^+^RGCs). **F–Y**: Representative isodensity maps showing the retinal distribution of RGCs in SD (albino) and PVG (pigmented rats). **F–I:** fluorogold traced RGCs, **J–M**: Brn3a^+^RGCs, **N–Q**: Brn3b^+^RGCs, **R–U**: Brn3c^+^RGCs, **V–Y:** Brn3^+^RGCs. For each marker and strain is shown the distribution of RGCs in one left and one right retina. Notice that, because FG and Brn3a or Brn3b and Brn3c were double detected, maps F&J, G&K, H&L, I&M, N&R, O&S, P&T and Q&U come from the same retinas. Isodensity maps are created from the data gathered after automated quantification. At the bottom of each one is shown the number of RGCs counted in the retina wherefrom the map has been generated. These maps express the RGC density according to a colour scale (bottom right in **I, M, Q, U** and **Y**) that ranges from 0 RGCs/mm^2^ (blue) to a maximum density (red) that is 3,200 RGCs/mm^2^ or more for FG^+^, Brn3a^+^and Brn3^+^RGCs; 1,800 RGCs/mm^2^ or more for Brn3b^+^RGCs, and 1,600 RGCs/mm^2^ or more for Brn3c^+^RGCs. The maximum density was adjusted to these numbers to allow the visualization of high and low density areas within the retina. Retinal orientation is shown in F–I: superior (S), nasal (N) temporal (T) and inferior (I). *Bars:* 20 µm (A), 1 mm (F, H).

Isodensity maps show the distribution of a given cell in the retina. In each of the frames captured per retina, densities are calculated from the data collected after the automated quantification. In **figure 4** is shown the distribution of FG-traced, Brn3a^+^, Brn3b^+^, Brn3c^+^and Brn3^+^RGCs in both rat strains. Because Brn3b and Brn3c populations are smaller than Brn3a, FG or Brn3, their density was set to a lower scale, otherwise the distribution of these RGCs would not be observed. Thus, red (highest densities) in the Brn3a, FG or Brn3 maps correspond to 3,200 or more RGCs and in the Brn3b and Brn3c ones to 1,800 and 1,600 or more RGCs, respectively.

In both strains all RGC populations are similarly distributed: they are denser in the medial-central retina and scarcer in the periphery. FG-traced and Brn3a^+^RGCs are densest in the superior temporal pole above the optic nerve while Brn3b^+^and Brn3c^+^RCGs although being denser in the central retina, are not clearly densest in the superotemporal quadrant.

PVG isodensity maps have cooler colours (lower RGC densities) than SD ones, in spite of the fact that there are more RGCs in this strain. This is because PVG retinas have a significantly bigger area (t-test p<0.001) than SD retinas (see **table 1**), thus their mean RGC density is lower.

### Population of Ipsilateral and Contralateral RGCs

In rats and mice the vast majority of RGCs project to the superior colliculi [18], [19], [22], [23], [46], [47]. To find out how many of them project to the contralateral or the ipsilateral SCi, FG was applied only to one SCi.

**Figure 5:**
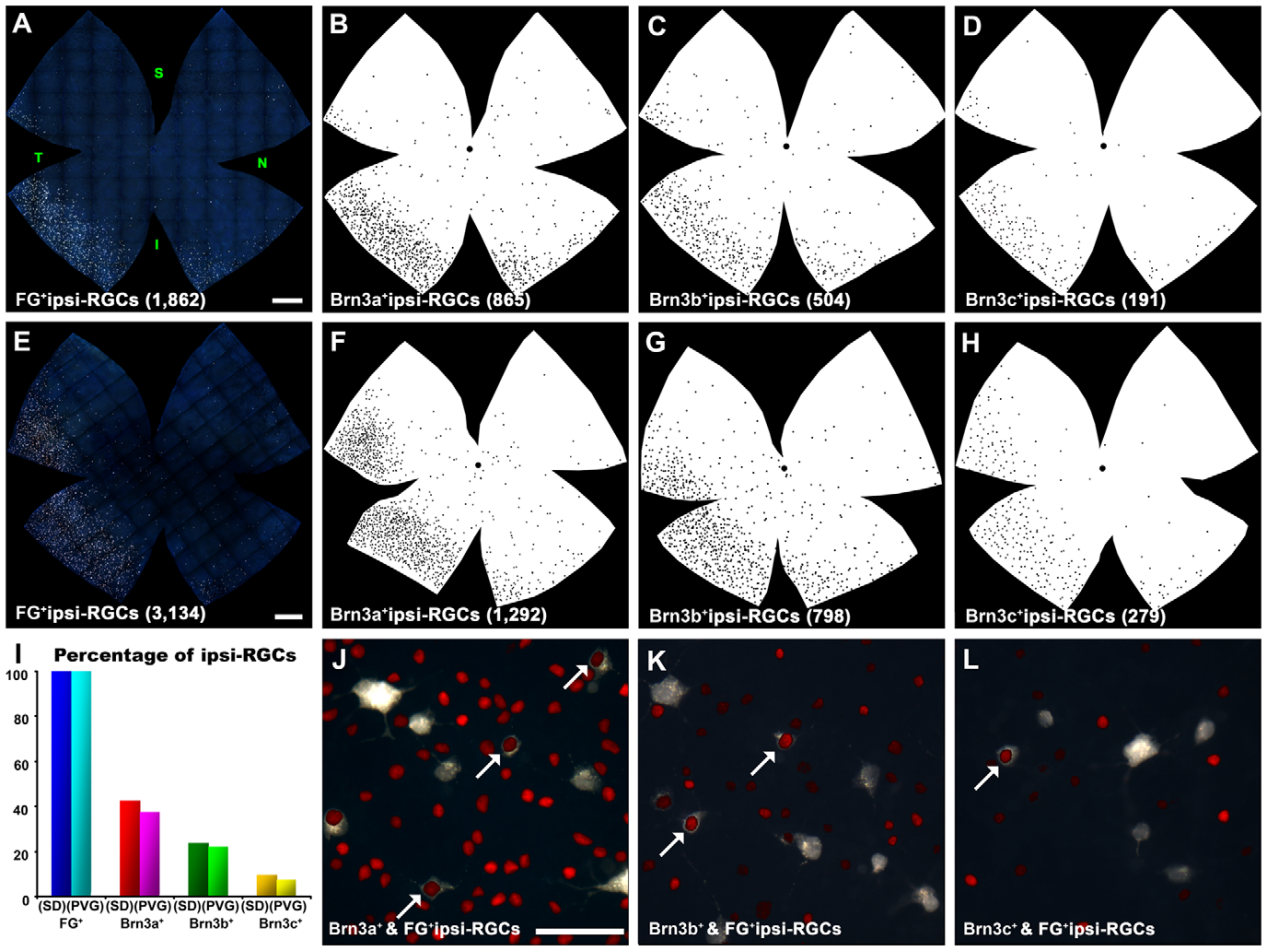
Distribution of ipsilateral RGCs in albino and pigmented rats. **A, E** Photomontages of an albino (**A**) and a pigmented (**E**) retina showing RGCs traced from the ipsilateral colliculi (ipsilateral-RGCs). **B–D, F–H:** retinal silhouettes showing the distribution of Brn3a^+^ipsilateral-RGCs **(B, F**), Brn3b^+^ipsilateral-RGCs (**C, D**) and Brn3c^+^ipsilateral-RGCs (**D, H**) in albino (**B–D**) and pigmented (**F–H**) rats. At the bottom of each retina is shown its number of ipsilateral-RGCs (in brackets). **I:** Histogram showing the percentage of Brn3a, Brn3b and Brn3c positive ipsilateral-RGCs with respect to their total number. **J–L:** Magnifications from the inferotemporal quadrant of ipsilaterally-traced retinas showing ipsilateral-RGCs and Brn3a (**J**), Brn3b (**K**) and Brn3c (**L**) RGCs. Arrows point to ipsilateral-RGCs that are Brn3 positive. Retinal orientation is shown in A: superior (S), nasal (N) temporal (T) and inferior (I). *Bars:* 1 mm (A,E), 50 µm (J).

**Table 2** shows the total number of contralateral- and ipsilateral- RGCs in both rat strains. Out of the total of RGCs that project to the SCi (i.e RGCs traced from both SCi, see **table 1**) 2.5% and 4.2% are ipsilateral-RGCs in albino and pigmented rats, respectively. Of these, 43% (SD) and 37.6% (PVG) express Brn3a, 24% (SD) and 22% (PVG) express Brn3b and 9.7% (SD) and 7.3% (PVG) express Brn3c. This means, without considering co-expression in this RGC population, that at least there is a 23.7% (SD) and a 33% (PVG) of ipsilateral-RGCs that do not express Brn3 (**Figure 5** **I–L and** **table 2**).

**Table 2:** Total number of contralateral and ipsilateral RGCs in albino and pigmented rats.

			Total number of RGCs				
			Contralateral	Ipsilateral*	Ipsilateral Brn3a^+§^	Ipsilateral Brn3b^+§^	Ipsilateral Brn3c^+§^
**SD**	**Mean±SD**		80,049±3,713	2,064±264	880±33	493±38	201±28
	**n**		14	14	5	4	5
**PVG**	**Mean±SD**		79,725±3,455	3,548±497	1,334±40	779±26	261±17
	**n**		9	9	3	3	3

Mean number ± standard deviation (SD) of contralateral-RGCs (left retinas) and ipsilateral-RGCs (right retinas) in the albino (SD) and pigmented (PVG) rats. It is shown as well the number of ipsilateral-RGCs that express Brn3a, Brn3b or Brn3c. n = number of analyzed retinas. *The pigmented strain has significantly more ipsilateral-RGCs than the albino one (t-test p<0.001). ^§^In the albino strain there are significantly more ipsilateral-RGCs that express Brn3a, Brn3b or Brn3c than in the pigmented one (t-test p<0.001).

Considering as 100% the total population of Brn3 a-, b- or c- positive RGCs, there is, respectively, a 1.06%, 0.91%, 0.48% in the albino strain and a 1.57%, 1.41% or 0.63% in the pigmented strain of Brn3a^+^, Brn3b^+^and Brn3c^+^RGCs that project ipsilaterally (**Figure 5I**).

Distribution of ipsilateral-RGCs in the right retinas is shown in **figure 5**. These cells are more abundant in the inferotemporal quadrant adopting a distribution that has the shape of a crescent moon going from the superotemporal to the inferonasal quadrant (**Figure 5** **A, E)**. Within the rest of the retina they are scarce and distributed without any apparent organization. This distribution is the same for ipsilateral-RGCs expressing Brn3a, b or c (**Figure 5** **B–D, F–H)**.

### RGC Nuclear Areas and Expression of Brn3

**Figure 6:**
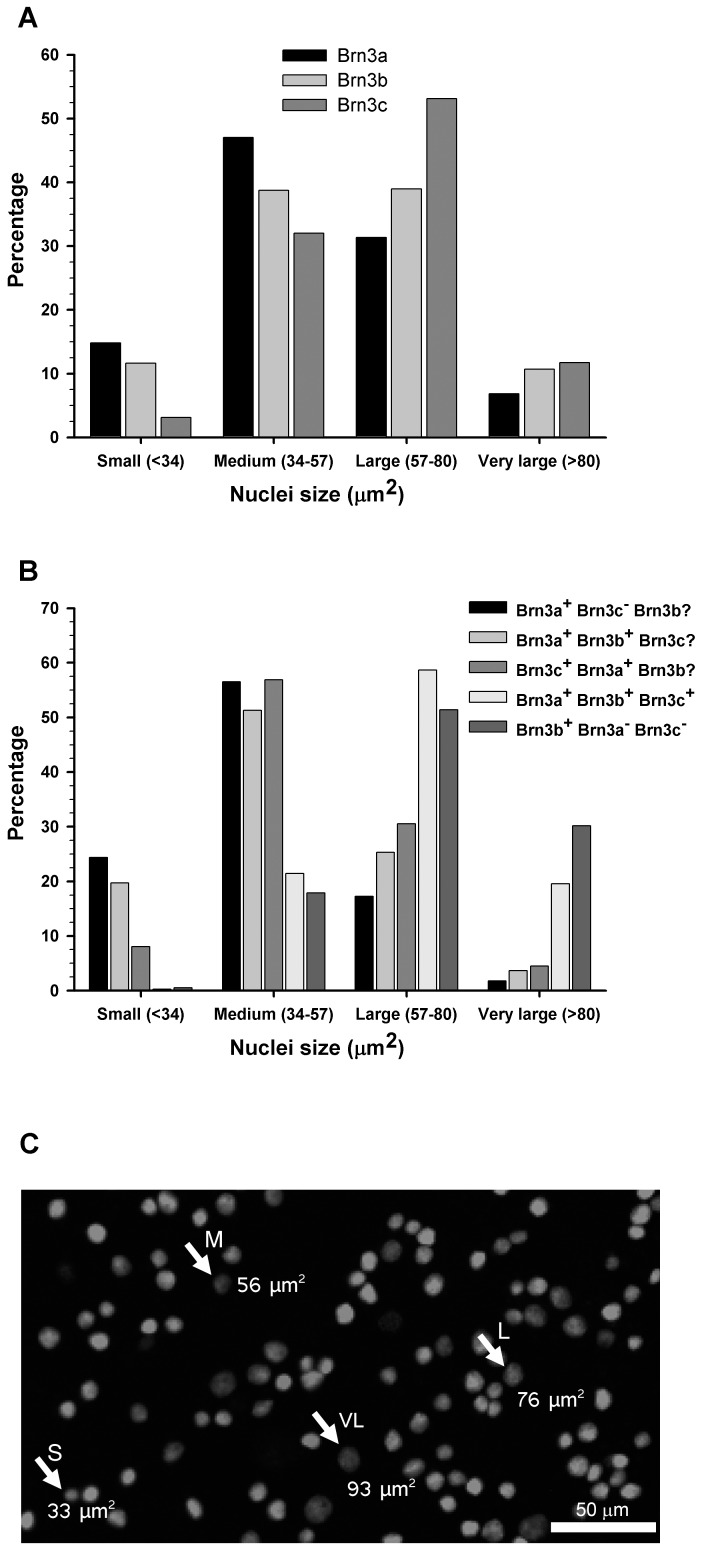
RGC nuclear sizes and expression of Brn3 factors. **A**: Histogram showing the percentages of Brn3a, Brn3b or Brn3c positive nuclei that are small, medium, large or very large. **B**: Histogram showing for each Brn3 combination the percentage of small, medium, large and very large nuclei (each Brn3 combination was considered 100%). **C**: Brn3a immunodetection showing the different sizes of RGC nuclei. An example of each size range is shown (S: small, M: medium, L: large, VL: very large).

In rodents, RGCs can be classified according to the pattern of their dendritic arbour, their soma size, projections to the brain and physiology (reviewed in [17]) and in, some cases, by molecular markers [24], [26]. Brn3 nuclear expression impedes classical morphological classification. However, from the retinal magnifications (see **figure 1** and **figure 6C**) it was clear that RGC nuclei have different sizes. Thus, the next goal was to measure their area having into account their Brn3 expression (**Figure 6**). RGC nuclei were grouped into small, medium, large and very large. Majority of nuclei are comprised between 34 and 89 µm^2^ (**Figure 6A**). While all nuclear sizes are represented in all Brn3 expression patterns (**Figure 6B**), some differences were observed: i) The major proportion of small nuclei corresponds to Brn3a^+^ones that do not express Brn3c; ii) Brn3a and Brn3b or Brn3c co-expression is more frequent in medium sized nuclei; ii) Nuclei that show triple Brn3 expression or expression of Brn3b only, are the biggest; iii) In accordance, the percentage of small nuclei expressing only Brn3b or that are triple positive is insignificant (0.3 and 0.5% respectively).

### Response of Brn3b and Brn3c to Optic Nerve (ON) Injury

**Figure 7:**
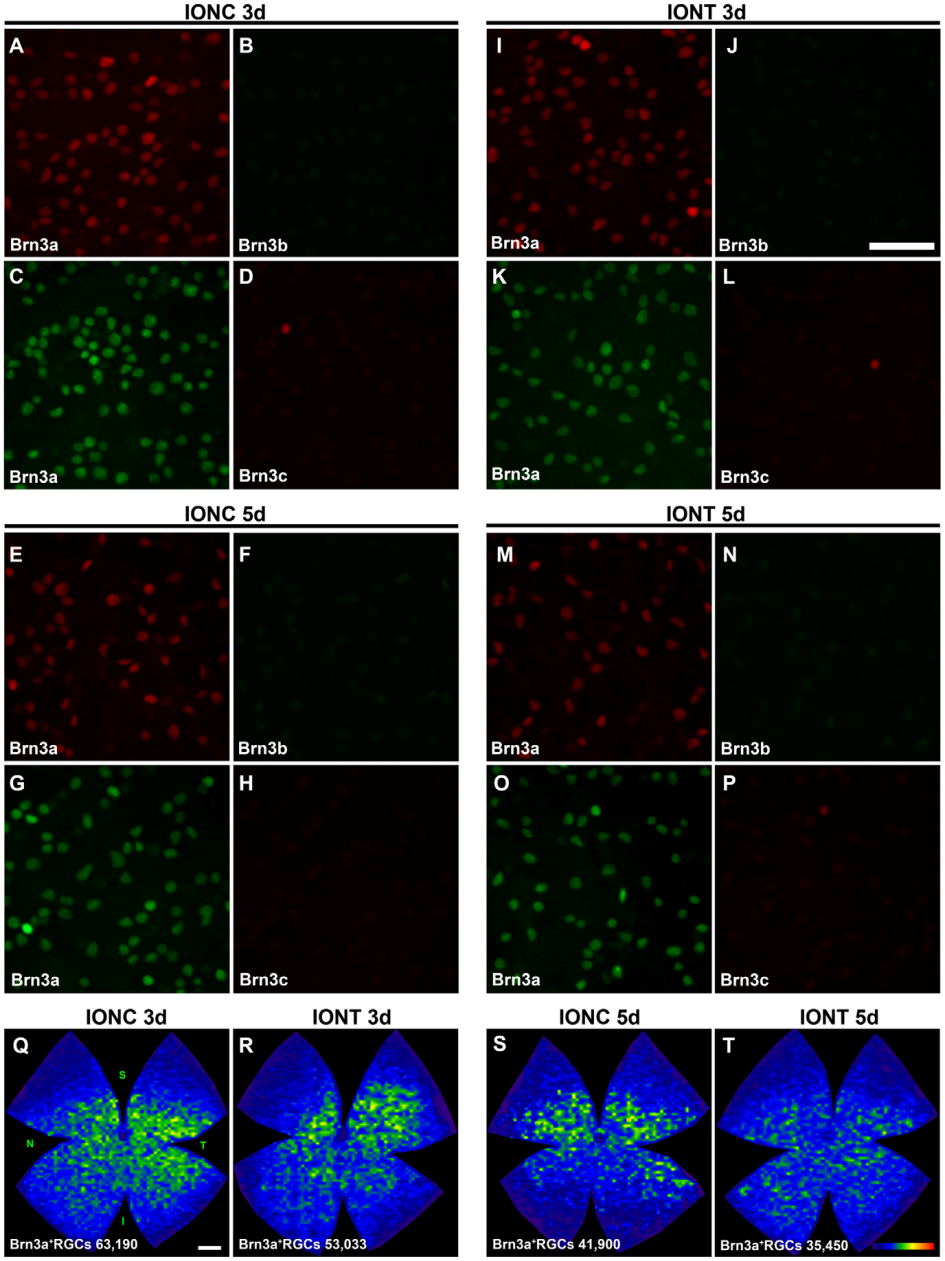
Brn3b and Brn3c expression is rapidly lost after optic nerve injury. **A-P**: Magnifications from SD flat mounted retinas analyzed 3 and 5 days after intraorbital nerve crush (**A–H**) and intraorbital nerve transection (**J–P**). As internal control, Brn3a was double immunodetected with Brn3b (**A–B, E–F, I–J, M–N)** or with Brn3c (**C–D, G–H, K–L, O–P**). In these images is observed that Brn3b and Brn3c expression decreases already 3 days after both injuries. This low signal impeded the automated quantification of Brn3b and Brn3c positive RGCs. Brn3a^+^RGCs were counted in these retinas and isodensity maps showing the distribution of the surviving RGCs were created (**Q–T**, at the bottom of each map is shown the number of Brn3a^+^RGCs counted in the retina wherefrom the map has been generated). These maps illustrate that IONT induces a quicker loss of RGCs than IONC, as evidenced by the higher densities observed in **Q** and **S** than in **R** and **T**. Colour scale (T bottom right) is the same as in figure 4M. Four (IONC 5d) or five (rest of the groups) retinas were analyzed per marker. *Bars:* 50 µm (J), 1 mm (Q).

To learn the effect of ON injury on Brn3b and Brn3c expression, three and five days after intraorbital optic nerve transection (IONT) or intraorbital optic nerve crush (IONC), these transcription factors were immunodetected. As internal control, Brn3a was detected as well (**Figure 7** **A–P**). These images show that Brn3b and Brn3c expression decreased below the sensitivity of our automated routine, while Brn3a signal was strong enough to run the automated quantification and to generate isodensity maps of the surviving Brn3a^+^RGCs (**Figure 7** **Q–T**). Thus, in these retinas the number of surviving Brn3a^+^RGCs was 60,691±3,653 or 52,684±3,099 (mean±SD) 3 days after IONC or IONT respectively and 40,555±3,932 or 30,114±3,116 5 days after IONC or IONT, respectively. At both time points, RGC loss was significant compared to the number of Brn3a^+^RGCs present in control retinas (Tukey t-test p<0.001). There is also a significant decrease between 3 and 5 days in both lesion models (Tukey t-test p<0.001). Finally, at both time points the loss of Brn3a^+^RGCs after IONT is significantly higher than after IONC (Tukey t-test, p = 0.005 at 3 days, p<0.001 at 5 days). This is graphically observed in the isodensity maps shown in **figure 7** **Q–T**.

**Figure 8:**
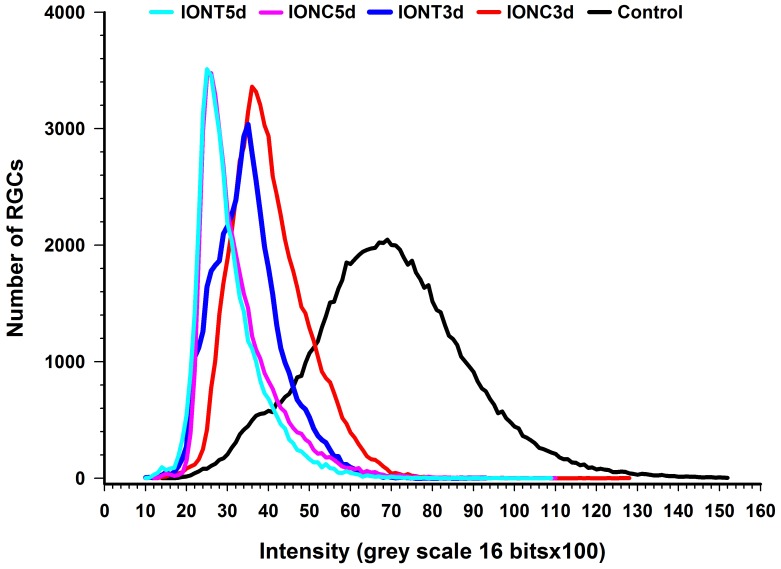
Injured RGCs down-regulate Brn3a expression. Plot depicting the distribution of RGCs according to their expression level of Brn3a. The number of RGCs (ordinate axis) is plotted against the intensity of their Brn3a signal in control, IONC and IONT injured retinas at 3 and 5 days post-lesion.

Brn3a signal is detected in injured retinas but, do RGCs down-regulate this transcription factor when injured? In other words, is there a decrease of Brn3a level in the injured but still alive RGC? In **figure 8** is shown that the mean intensity of Brn3a signal decreases already at 3 days post-lesion, being this decrease slightly more pronounced after IONT than after IONC. By 5 days post lesion the level of Brn3a signal has decreased further and at this time point both lesions are matched.

## Discussion

Here we present the first comprehensive analysis of the expression of the three Brn3 members of transcription factors in albino and pigmented rat retinas.

With respect to Brn3 differences between albino and pigmented rats, we have found that in both strains their number, proportion, co-expression, and distribution are similar. Thus discussion of these data refers to both strains. The only significant difference found between both strains is in the ipsilateral-projections, this is further addressed below.

### Identification of Retinal Ganglion Cells

Neuronal tracers have been used extensively to identify RGCs in control and injured retinas [48]–[52], although the lesion involves spurious labelling of microglial cells [53]–[56], making it difficult to ascertain RGC survival. There are a number of molecular markers that are associated with RGCs, such as γ-synuclein, Bex1/2, Thy1, NeuN, or Brn3a [39], [57]–[63]. Currently, two of these markers stand out: detection of γ-synuclein RNA [61]–[63] and immunodetection of Brn3a [39]. Brn3a immunodetection is an ex vivo method that can be easily set up in any laboratory to detect, quantify, and assess the spatial distribution of RGCs in health, disease and neuroprotection [64]–[72].

Previously, we documented that in rat, Brn3a is expressed in 92.2% of the FG-traced RGC population [39]. So, which RGCs are not being detected by Brn3a? Data presented here show that Brn3a is expressed in 43% (SD) and 38% (PVG) of the ipsilateral population, and by a minute proportion of the intrinsically photosensitive RGCs. In other words, when using Brn3a to identify RGCs, the RGC sub-populations that are not being detected are half of the ipsilateral-RGCs and all of the ip-RGCs.

### Brn3 Co-expression, Total Numbers and Distribution

In the pigmented strain the total number of RGCs is higher than in the albino one, although this difference is not significant.

The more abundant Brn3 member is Brn3a which is expressed by the vast majority of FG-traced RGCs. So, with respect to Brn3a, Brn3b is expressed by 65% (SD and PVG) of the total RGC population, while Brn3c is expressed by 51% (SD) and 49% (PVG) of them. Thus, while the pigmented strain has more RGCs than the albino one, the proportions that express each Brn3 member are maintained.

The level of Brn3 co-expression reaches 70% of the RGC population. Out of this 61% of the RGCs express the three members (37% of the total population) while RGCs that only express one Brn3 are mainly Brn3a^+^(26%) being the rest Brn3b^+^.

In mouse there is not such a detailed study. Gathered from Badea’s et al work [4] there are two main differences between both species: i) in mouse 20% of Brn3c^+^RGCs do not express Brn3a, and ii) the level of Brn3b and Brn3c co-expression is over 90%. These data are slightly different from those of Xiang et al [5] that reported that 36% of the cells in the mouse ganglion cell layer (GCL) were Brn3a^+^and 35% were Brn3b^+^while a 37% were labeled when both proteins were detected at the same time. Brn3c only labeled 15% of neurons in the GCL, and when it was combined with Brn3b or Brn3a the percentage of detected nuclei was close to that observed with Brn3a or Brn3b only. Because in the GCL approximately one half of the neurons are RGCs [73], [74] then, a 72% of RGCs in mice express Brn3a, 70% Brn3b and 30% Brn3c. In addition, their data also show that most of the mouse RGCs co-express Brn3a and Brn3b, and that all Brn3c RGCs are a subset of those expressing Brn3a and Brn3b.

All these data prompt to speculate that because in mouse Brn3b is the first Brn3 to be expressed and it is needed for RGC differentiation and survival then, most adult RGCs in this species would continue expressing Brn3b. Conversely, in the adult rat retina Brn3a is the dominating Brn3 factor and it might replace Brn3b in its differentiation and survival role. However, to support this notion two sets of experiments should be addressed: quantification of the total population of mice RGCs Brn3b and Brn3c positive (Brn3a population has been already described, [38]) and a developmental analysis of Brn3 expression in rat.

The topography of FG-traced and Brn3a positive RGCs in the adult rat [23], [39] and mouse [22], [38], [75] retina has been previously described. In both species, RGCs are denser in the medial-central retina, where the highest densities are located above the optic nerve along the nasotemporal axis, peaking in the temporal quadrant [75]–[77]. This area has been proposed to be the visual streak of rats and mice [22], [23], [78]. This idea is reinforced by the fact that, at least in rats, L-cones reach their highest densities in the same location as RGCs [78].

Distribution of Brn3b or Brn3c positive RGCs resembles that already described, in the sense that majority of them are found in the medial-central retina. However, while in the temporal retina their densities are higher they do not group around the visual streak as Brn3a^+^RGCs do. Thus, in the visual streak, the region of the retina specialized to provide the best vision at some point in the visual space, most of the RGCs are those that solely express Brn3a.

### Intrinsically Photosensitive RGCs

Few ip-RGCs express Brn3, in fact none of them express Brn3c and around 0.20% of them express Brn3a. With respect to Brn3b the percentage is close to 10% in the albino and to 14% in the pigmented strain. This scenery differs significantly from mouse where 65% of them express Brn3b [27], [79], [80]. In rat it has been shown that ip-RGCs are more resilient to axonal injury and glaucomatous damage than the rest of RGCs [81], [82]. Because both, Brn3a and Brn3b are linked to RGC survival (see below) it is possible that ip-RGCs resilience is due to their independence of Brn3 factors. However, to further support this suggestion it should be investigated whether the ip-RGCs that die after injury are those that express Brn3b.

### Retinal Ganglion Cells Projecting Ipsilaterally

The only difference observed between strains was in the numbers and proportion of the ipsilateral population, a fact that it is known since the 60′s [19], [46], [83]–[86] and that explains the poorer binocular vision of albino animals [41]. Whether the crucial difference lies in their numbers or in their proportion, it is something that requires further research. Here, we complement these studies reporting their total numbers and their expression of Brn3. Two novel conclusions can be drawn from these data: i) because the contralateral population is almost the same in both strains (see tables 2 and 3), the higher numbers of RGCs counted in pigmented rats seems to be due to ipsilateral-RGCs; ii) more ipsilateral-RGCs express Brn3s in the albino than in the pigmented rats.

The distribution of ipsilateral-RGCs in rats matches that previously described in rodents [19], [21], [46], [77], [87]–[89]. These cells are scattered throughout the retina except in the temporal region, where they are densest in the periphery and distributed in the shape of a crescent moon that goes from the ventral to the dorsal retina. But, as shown in albino and pigmented mice [19], in the pigmented rat ipsilateral-RGCs are homogenously dense in this crescent region, while in the albino strain there is a decrease of these cells towards the central-dorsal retina.

### RGC Classes

Data presented here are insufficient to determine RGC classes based on their somatic size, dendritic arborizations, physiology or projections [17], [21], [37]. However, if we assume that the bigger the nucleus the bigger the soma, those RGCs that express the three Brn3 members or that only express Brn3b are large/very large, while those that express Brn3a and/or Brn3b or Brn3c are medium/small. These data can be correlated with those by Dreher et al [21]. These authors show that RGCs that project to the dorsal lateral geniculate nuclei (DLG) have bigger somas than those projecting to the superior colliculi (SCi). Thus, it is possible that RGCs expressing the three Brn3s are those projecting to the DLG, while those that co-express two members project to the SCi. In addition, nuclei that only express Brn3b are the biggest ones, they might correspond to Class I ganglion cells [21] and to ip-RGCs [37].

### Brn3 Response to Axonal Injury

We have previously shown that Brn3a expression is maintained in RGCs as long as they are alive, irrespectively of being injured [40]. We wanted to know whether this was also true for Brn3b and Brn3c, but we were unable to determine it, because after optic nerve injury their level of expression decreased below the signal needed for quantification. This does not necessarily reflect that their expression is lost, only that is diminished, since as shown in figure 6 some very faint positive nuclei are observed at 3 and 5 days post-lesion. In these same retinas, Brn3a was immunodetected and it was observed that three days after both lesions there was already a significant loss of RGCs followed by a further significant loss at 5 days.

RGC loss itself explains the reduction of Brn3a in the retina, but is this decrease due as well to its down-regulation in the surviving RGCs? Analysis of the signal intensity of Brn3a shows that already at 3 days post-IONT or IONC the surviving RGCs have a lower than control Brn3a signal. Furthermore, the mean intensity of the IONT plot at this time point is lower than the IONC one, which agrees with the fact that optic nerve transection is a more severe injury than optic nerve crush [39], [51], [90], [91]. This is important because Brn3a has been shown to have anti-apoptotic effects [92]–[97], and Brn3b has a role in rat RGC survival [98]. It is therefore tempting to speculate that their quick and continuous down-regulation by the axotomized RGCs [39], [59] may be one of the multiple molecular signals that cause their death [99], [100] instead of a consequence.

## Materials and Methods

### Animal Handling, Anaesthesia and Analgesia

Adult female albino Sprague-Dawley (SD, 180–220 g body weight) and pigmented Pievald Virol Glaxo (PVG, 220–250 g body weight) rats were obtained from the University of Murcia breeding colony. All experimental procedures were carried out in accordance with the Association for Research in Vision and Ophthalmology and European Union guidelines for the use of animals in research. This study was approved by the Ethics Committee for Animal Research of the University Hospital Virgen de la Arrixaca (Comité Ético de Experimentación Animal (CEEA) del Hospital Universitario Virgen de la Arrixaca, Murcia, Spain).

In the animals groups subjected to surgery for anaesthesia a mixture of xylazine (10 mg/kg body weight; Rompun®; Bayer, Kiel, Germany) and ketamine (60 mg/kg body weight; Ketolar®; Pfizer, Alcobendas, Madrid, Spain) was used intraperitoneally (i.p.). After surgery, an ointment containing tobramicin (Tobrex; Alcon S.A., Barcelona, Spain) was applied on the cornea to prevent its desiccation. Rats were given oral analgesia (Buprex, Buprenorphine 0.3 mg/mL, Schering-Plough, Madrid, Spain) at 0.5 mg/kg (prepared in strawberry-flavoured gelatine) the day of the surgery and during the next 3 days.

All animals were sacrificed with an i.p. injection of an overdose of pentobarbital (Dolethal, Vetoquinol®, Especialidades Veterinarias, S.A., Alcobendas, Madrid, Spain).

### Surgery

#### RGC tracing from both superior colliculi

Fluorogold (FG, 3% diluted in 10% DMSO-saline, Fluorochrome, LLC, USA) was applied to both superior colliculi (SCi) in albino and pigmented rats one week prior animal processing, following standard techniques in our laboratory [39], [51], [66], [67], [90].

#### Tracing of RGCs projecting ipsilaterally

In both rat strains, the left SCi was removed by aspiration. One week later Fluorogold was applied to the right SCi and animals were processed a week later.

#### Optic nerve (ON) axotomy

In albino SD rats, the left ON was intraorbitally injured according to standard procedures in our laboratory [39], [51], [52], [90]. For intra-orbital optic nerve transection (IONT), the ON was sectioned 0.5 mm from the optic disc, while for intra-orbital optic nerve crush (IONC) the ON was crushed during 10 seconds at the same distance from the optic disc using watchmaker’s forceps.

### Experimental Design

#### Flat mounted retinas from untouched albino and pigmented rats

-Double immunodetection of: i) Brn3a and Brn3b, ii) Brn3a and Brn3c; iii) Brn3b and Brn3c; iv) melanopsin and Brn3a; v) melanopsin and Brn3b; vi) melanopsin and Brn3c. These groups were prepared to assess the percentage of Brn3 co-expression and the population of intrinsically photosensitive RGCs (ip-RGCs) that express each member of the Brn3 family. Group iii was used, as well, to quantify the whole population of Brn3b or Brn3c positive RGCs.

-Double immunodetection of mouse anti-Brn3a and goat anti-Brn3a. This group was prepared to check if both antibodies recognized the same RGC population.

-Triple immunodetection of Brn3a, Brn3b and Brn3c, detecting all of them with the same fluorophore. This group permitted to quantify the whole number of Brn3 positive RGCs (Brn3^+^RGCs).

#### Retinal cross sections from untouched albino rats

Single immunodetection of Brn3a, Brn3b and Brn3c.

#### Flat mounted retinas from albino and pigmented rats traced from both superior colliculi

FG-tracing from both SCi labels 98% of RGCs in albino and pigmented rats [23]. Brn3a immunodetection in albino rats detects 92.2% of the FG-traced population [39]. This group was used to quantify the whole number of Brn3a^+^RGCs and of RGCs traced from both SCi (henceforth FG-traced RGCs) in both rat strains, and served as baseline to calculate the proportion, with respect to the total RGC population, of RGCs that express Brn3b or Brn3c.

#### Flat mounted retinas from albino and pigmented rats traced from one superior colliculi

Retinas ipsilateral to the labelled colliculus (right) were used. In these, Brn3a, Brn3b and Brn3c were single immunodetected. With this group it was quantified the whole population of RGCs that project ipsilaterally (henceforth ipsilateral-RGCs) and assessed the expression of the three Brn3 members in this RGC subpopulation. In addition, the total number of FG-traced RGCs in the left retina (contralateral-RGCs) was quantified as well.

#### Flat mounted retinas from albino rats subjected to optic nerve injury

In these retinas Brn3a and Brn3b or Brn3a and Brn3c were double immunodetected.

The number of retinas analyzed per group and analysis ranged from 6 to 12 except for ON injured retinas and ip-RGCs (n = 4−5 per analysis, injury and time point). For details see results (table 1, table 2 and figure 3 legend).

### Retinal Dissection

Animals were perfused transcardially with 4% paraformaldehyde (PFA) in phosphate buffer 0.1M after a saline rinse.

Right after deep anesthesia and before fixation a suture was placed on the dorsal pole of each eye. Retinas were dissected as flattened whole-mounts by making four radial cuts (the deepest one in the dorsal pole), post-fixed for an additional hour in 4%PFA and kept in phosphate buffered saline (PBS) till further processing. For cryostate sectioning, dissected eyes were cryoprotected in 30% sucrose (Sigma, Alcobendas, Madrid, Spain) before embedding them in optimal cutting temperature (OCT) compound (Sakura Finetek, Torrance, CA).

### Immunohistofluorescence Protocol

Antibody list and working dilutions are shown in **table 3**.

**Table 3:** Antibodies and working dilutions.

Primary antibodies	Dilution	Cat number and Company
Goat anti-Brn3a	1∶750	sc-31984, Santa Cruz Biotechnologies. Heidelberg, Germany
Mouse anti-Brn3a	1∶50	sc-8429, Santa Cruz Biotechnologies.Heidelberg, Germany
Goat anti-Brn3b	1∶50	sc-31989, Santa Cruz Biotechnologies.Heidelberg, Germany
Mouse anti-Brn3c	1∶250	sc-81980, Santa Cruz Biotechnologies.Heidelberg, Germany
Rabbit anti-melanopsin	1∶500	PA1-781, Affynity Bioreagents. Fisher Scientific, Madrid, Spain
**Secondary antibodies**		
Donkey anti-goat Dylight 594	1∶500	Jackson ImmunoResearch, Newmarket, Suffolk, UK
Donkey anti-goat Alexa 488		Molecular Probes, Life Technologies, Madrid, Spain
Donkey anti-rabbit Alexa 488		
Donkey anti-rabbit Alexa 594		
Donkey anti-mouse Alexa 594		
Donkey anti-mouse Alexa 488		

#### Flat mounted retinas

Whole mounts were permeated in PBS 0.5% Tritonx100 (Sigma Aldrich Química, Madrid, Spain) by freezing them during 15 minutes at −70°C, rinsed in new PBS 0.5%Triton and incubated overnight at 4°C with the appropriate antibody combination diluted in blocking buffer (PBS, 2% normal donkey serum, 2% Triton). Afterwards, retinas were washed three times in PBS-0.5% Triton and incubated 2 hours at room temperature with fluorescence conjugated-secondary antibodies diluted in blocking buffer. Finally, after thorough washing in PBS-0.5% Triton, retinas were rinsed in PBS, mounted vitreal side up and covered with anti-fading solution (Vecta-Shield Mounting Medium, Vector Laboratories, Alicante, Spain).

#### Retinal cross-sections

Sections (15 µm) were washed 3 times with PBS to eliminate the OCT; then, sections were incubated overnight at 4°C with the appropriate antibodies diluted in blocking buffer (PBS, 2% donkey normal serum, 0.1% Tritonx100). Next day, sections were washed with PBS-0.1% Triton and incubated 1 h at room temperature with fluorescence conjugated-secondary antibodies diluted in the same blocking buffer. Finally, after thorough washing in PBS-0.1% Triton, sections were rinsed in PBS and mounted with anti-fading Vecta-Shield Mounting Medium with DAPI (Vector Laboratories, Alicante, Spain).

### Image Acquisition

All samples were photographed with an epifluorescence microscope (Axioscop 2 Plus; Zeiss Mikroskopie, Jena, Germany) equipped with a computer-driven motorized stage (ProScan™ H128 Series, Prior Scientific Instruments, Cambridge, UK), controlled by the Image Pro Plus software, (IPP 5.1 for Windows®; Media Cybernetics, Silver Spring, MD, USA), as previously described [23], [39], [78]. Briefly: to make reconstructions of retinal whole-mounts, retinal multi-frame acquisitions were taken for each fluorophore in a raster scan pattern. Frames were captured contiguously side-by-side with no gap or overlap between them using an ×10 objective (Plan-Neofluar, 10×/0.30; Zeiss Mikroskopie, Jena, Germany). The images taken for each retina were saved in a folder as a set of 24-bit colour image pictures. Importantly, for each Brn3 member the acquisition settings were the same for all retinas in order to carry out the analysis of Brn3 expression level detailed below. Later, these images were combined automatically into a single tiled high resolution composite image of the whole retina using IPP® for Windows®.

### Co-expression Analyses

Magnifications from flat mounted retinas were taken acquiring, for the same frame, the signal of each detection combination (i.e.: Brn3a+Brn3b, Brn3b+Brn3c and so on). Then, images were coupled using Adobe Photoshop. Red or green signal meant expression of one of the markers, and yellow co-expression. Using a minimum of 16 frames taken randomly from 4 different retinas per strain and marker, it was quantified the number of RGCs or ip-RGCs expressing each Brn3 combination.

### RGC Ipsilateral and Contralateral Population

In the animals in which FG was applied to the right superior colliculi, FG-traced RGCs in the left retinas are those projecting contralaterally (contralateral-RGCs) and in the right retinas those projecting ipsilaterally (ipsilateral-RGCs).

Retinas were photographed as above-mentioned. In the left and right retinas FG signal was acquired and automatically quantified (see below). In addition in the right retinas Brn3a, Brn3b or Brn3c were single immunodetected and photographed. The resultant photomontages of ipsilateral-RGCs and Brn3a or Brn3b or Brn3c were coupled with Adobe Photoshop. In a new layer, the position of the ipsilateral-RGCs positive for Brn3a, Brn3b or Brn3c was dotted. Dots were quantified to have the numbers of ipsilateral-RGCs that express each Brn3 member and, to show their position in the retina, the retinal silhouette with the dots was cropped.

### Automated Quantification of the Total Population of RGCs

Brn3a and FG-traced RGCs were quantified using previously reported automated routines [23], [39]. The Brn3a automated counting method was suitable to quantify as well Brn3^+^RGCs.

However, to quantify the whole population of Brn3b and Brn3c positive RGCs, we developed two specific subroutines. The IPP macro language was used to apply a sequence of filters and transformations to each image to clarify cell limits and to separate individual cells for automatic cell counting. In a first step, images were filtered with a Despeckle (5×5) filter to reduce impulse noise. This step was followed by the application of the filter Open (kernel size 3×3) to smooth object contours, separate narrowly connected objects and remove small dark holes. The resultant image was then filtered with a Flatten filter, which evens out background variations, reducing intensity variations in the background pixels. The resultant image data were converted 16-bit gray scale to discard the colour information. After that, the image was passed through the Median (3×3) filter to remove impulse noise, replace central pixels with median value in its neighbourhood. Then, the image data were filtered again with the large spectral Edge+(20) filter, which extracts positive edges of the objects. Cell clusters were separated by two passes of the IPP watershed split morphologic filter, which erodes and dilates objects separating overlapping ones. Positive nuclei were counted within predetermined parameters to exclude objects that were too large or too small to be RGCs nuclei. These parameters were also used to classify the objects into two levels of Brn3b or Brn3c signal, faint and strong. Finally, the user was asked to validate or adjust some counting parameters in order to control artefacts and avoid mismatches. Because this adjustment is a subjective factor introduced by the user, to avoid this influence all counts were repeated three times with a two week lapse between counts. Results from the 3 quantifications were compared using the SPSS (SPSS v11 IBM Corporation). The intra-class correlation coefficient (ICC) test value was ICC = 0.952. This result supports the strength of the method and dispels any subjective factor. As last step, data of each count were displayed and exported by dynamic data exchange to a spreadsheet (Office Excel 2007; Microsoft Corp., Redmond, WA) where the data were filed and saved for further analysis.

### Validation of the Brn3b and Brn3c Automated Counting Method

To validate the Brn3b and Brn3c automated counting routine, three different experienced investigators counted manually and in a masked fashion, a total of 7,818 Brn3b^+^RGCs and 6,920 Brn3c^+^RGCs present in 17 frames. These frames represented different RGC density regions and were randomly selected from seven whole-mounted retinas. These results were plotted against the counts obtained automatically (7,583 Brn3b and 6,630 Brn3c positive nuclei). The high correlation between both methods (Pearson correlation test, *R* ^2^ = 0.95 for Brn3b and *R* ^2^ = 0.96 for Brn3c) validates the automated method.

### Measurement of Brn3a Expression Intensity

As a last step of the previously described counting routines, a new transformation was performed by the creation of a black and white mask in which the counted nuclei were represented as white and the rest of the image as black. The original image was then multiplied by this new black and white mask and a new clean image, containing only the counted nuclei as they were in the original image (i.e. each nuclei with its original intensity), was produced. This image was then transformed into a 16-bit gray scale image where the signal intensity of each nucleus was measured in fluorescence arbitrary units, where white (maximum signal) was 65,535 and black (no signal) was 0. These values were exported to an Excel sheet and plotted using SigmaPlot 10.0 (Systat Software Inc).

### Measurement of Nuclei Area

In the magnifications from the albino strain used for the co-expression analysis, Brn3^+^nuclei area was measured. The perimeter of each nuclei was manually outlined and the area within automatically measured using the IPP software. Areas were collected from 8,494 nuclei; of these 8,315 were Brn3a^+^, 4,855 Brn3b^+^and 3,599 Brn3c^+^. Having into account Brn3 co-expression, 2,828 were Brn3a&Brn3b^+^, 1,329 Brn3a&Brn3c^+^, 1,848 Brn3b&Brn3c^+^(therefore also Brn3a^+^, see results), 2,310 Brn3a^+^and Brn3c^-^, and 179 Brn3b^+^, Brn3a^-^ and Brn3c^−^. Notice that in all cases of double expression, nuclei could also express the third Brn3 member, but because two fluorophores were used it was not possible to determine how many. In addition, this precluded as well the assessment of nuclei expressing only Brn3a.

Values were exported to an Excel sheet and plotted using SigmaPlot 10.0 (Systat Software Inc).

### Isodensity Maps

Detailed spatial distribution of the different populations of RGCs over the entire retinas was demonstrated with isodensity maps constructed as previously described [23], [39], with the exception that Brn3b and Brn3c densities were calculated in areas of interest of 0.0767 mm^2^ instead of 0.0074 mm^2^. Isodensity maps are created from the data gathered after automated quantification. With these maps is illustrated the RGC density and its distribution in the whole retina according to a colour scale that goes from 0 RGCs/mm^2^ to a maximum density that was set to: 3,200 RGCs/mm^2^ or more for FG^+^, Brn3a^+^and Brn3^+^RGCs, 1,800 RGCs/mm^2^ or more for Brn3b^+^RGCs and 1,600 RGCs/mm^2^ or more for Brn3c^+^RGCs. The maximum density was adjusted to these numbers to allow the visualization of high and low density areas within the retina.

### Statistics

Data were analyzed with Sigma Stats Ver. 3.11 (Systat Software Inc, 2004). Tests are detailed in results. Differences were considered significant when p<0.05.
